# 438. Predicting Severe and Critical COVID-19 in Fully Vaccinated Individuals: a Single Center Retrospective Cohort Study

**DOI:** 10.1093/ofid/ofad500.508

**Published:** 2023-11-27

**Authors:** Charles Kevin L Rivera, Mark B Carascal, Ma Cristina Perez, Raul V Destura, Karl Evans R Henson

**Affiliations:** The Medical City, Pasig City, National Capital Region, Philippines; The Medical City, Pasig City, National Capital Region, Philippines; The Medical City, Pasig City, National Capital Region, Philippines; The Medical City, Pasig City, National Capital Region, Philippines; The Medical City, Pasig City, National Capital Region, Philippines

## Abstract

**Background:**

The ability to predict severe/critical Coronavirus Diseases 2019 (COVID-19) infection in fully vaccinated individuals is an incompletely defined area of research and a scoring tool is lacking. Most scoring systems are validated against unvaccinated patients making it less clinically applicable in today’s global landscape. Our study validated an existing clinical prediction tool using data from vaccinated individuals, and created a new model that could provide improved prediction accuracy.

**Methods:**

Consecutive adult patients fully vaccinated against COVID-19 and admitted to a tertiary hospital from March 2021 to August 2022 for a breakthrough infection were enrolled. Clinical characteristics, type and number of vaccine doses, and RT-PCR cycle-threshold (CT) values were collected. Primary outcome of interest was development of severe/critical COVID-19. An existing severe/critical COVID-19 prediction tool was validated using study data as a comparator prediction tool. Multivariable logistic regression analysis was performed to create a simpler scoring system to predict COVID-19 severity.

**Results:**

Among 390 adults with COVID-19, 106 (27%) received at least 1 booster dose and 54 (14%) progressed to severe/critical disease. The existing prediction tool had a sensitivity (Sn) of 98.1%, specificity (Sp) of 7.5% and area under the receiver-operator curve (AUROC) of 0.58. Multivariable logistic regression showed that age >75y (OR 2.15; 95% CI 1.04 - 4.47), diabetes mellitus (OR 1.98, 95% CI 1.07-3.67), CT value < 20 (OR 3.48; 95% CI: 1.78 - 6.79), and non-receipt of a booster dose (OR 0.26; 95% CI: 0.11 - 0.64) were independent risk factors for developing severe/critical COVID-19. The final model showed a Sn of 65%, Sp of 68% and AUROC of 0.71.

**Conclusion:**

A simplified scoring tool using 4 variables may help predict progression to severe/critical COVID-19 in fully vaccinated individuals. Although the tool has modest Sn and Sp, it can be used to help triage patients and guide initial therapy. Validation of this tool in larger studies is recommended.

**Disclosures:**

**All Authors**: No reported disclosures

